# Two Successful Cases of Delivery by the Crotchet, in Extreme Deformity of the Pelvis

**Published:** 1786

**Authors:** John Clarke

**Affiliations:** Surgeon Man-midwife, in London


IX. *Two ſucceſsful Caſes of Delivery by the Crotchet, in extreme Deformity of the Pelvis. Communicated in a Letter to Dr.* Simmons, *by Mr.* John Clarke, *Surgeon Man-midwife, in London.*

I Take the liberty of communicating to you two caſes of laborious parturition, both of which fell under my own obſervation, and the laſt under my immediate care. As they tend to confirm ſtill farther the idea, that recourſe to the Cæſarean ſection is unneceſſary, even under circumſtances, of extreme deformity of the pelvis, which have been generally ſuppoſed to require it: and as the Cæſarean operation in this country has been ſo invariably fatal, you will perhaps think them not unworthy a place in the London Medical Journal.

Opinions

Opinions held forth and supported by men whofe fkill and abilities are highly efteemed, feldom fail to have very confiderable weight with mankind; and undoubtedly, by being derived from fuch fources, they have a reafonable claim to the attention of the public. We have, however, but too frequently reafon to lament, that in all cafes implicit confidence cannot be placed in them, becaufe they are fometimes not founded in truth. Errors in practice, however fupported, may prove detrimental to fociety; but the danger increafes in proportion to the reputation which thofe who propagate them hold in the eftimation of the world. I am led into this reflexion from confidering the ideas which have been adopted and publifhed, refpecting the practice which ought to be purfued where the pelvis is found at the time of labour to be peculiarly fmall. Profeffor Plenck, of Vienna, has ftated, that if we could be affured of the life of the child, in a cafe where the fmall diameter of the pelvis at its fuperior aperture is lefs than three inches, we ought to have recourfe to the Cæfarean fection, with the view of preferving the life of the child: but that if the child be dead, we may deliver it by leffening the bulk of the head.

Upon this part of his aſſertion it is unneceſſary to make any comment, becauſe, under ſuch circumſtances practitioners in this country, having accurately weighed the chance of the child's life againſt that of the mother, have, as far as I am informed, determined univerſally to deliver by the crotchet. But he proceeds to add, that even if we were convinced of the death of the child, in a caſe where the ſmall diameter is leſs than two inches, we muſt either leave the patient to die, or have recourſe to the Cæſarean ſection; becauſe, as he aſſerts, it is impoſſible in ſuch narrow dimenſions, to bring the head of a child at its full time through it, even when the head is leſſened by evacuating the brain.

To counteract the fatal tendency of ſuch a doctrine, ſupported by ſo high an authority, Dr. Oſborn has publiſhed a caſe*, where he delivered the patient under circumſtances infinitely more difficult, by leſſening the volume of the head. From the event of that caſe, he has concluded, that the head of a mature fœtus may, with perfect ſafety to the mother, be brought through a pelvis where the diameter is

* Vide Eſſay on laborious Parturition. 8vo. London, 1783.

equal equal only to an inch and a half, or even leſs, from the os pubis to the projecting angle of the os ſacrum. A number of experiments are however neceſſary in order to give due weight to any aſſertion. Fortunately for the eſtabliſhment of this, two caſes have occurred within a few months, which juſtify the opinion, and by their ſucceſs will probably change the ſentiments of a late author, who ſeems to think, that not more than four or five women out of fifty, where the ſmall diameter of the pelvis is leſs than two inches, can ſurvive the delivery by the crotchet.

### CASE I.

Ann Cooper, aged twenty-one, when ſhe was about two years old, became affected with the rickets, in conſequence of which ſhe was very much diſtorted, both in her ſpine and inferior extremities, ſo that ſhe only meaſures in height four feet and two inches. In the middle of October 1784, ſhe became pregnant. During the early part of her pregnancy ſhe had few complaints, but towards the end of it ſhe had a great deal of pain.

She was admitted into the Store-ſtreet hoſpital in the morning of Tueſday, July 19,

1785,

1785, and about five o'clock in the afternoon the os uteri began to dilate very ſlowly, but no part of the child could be felt. Her pains continued very ſtrong and frequent through the whole day, and ſhe vomited ſeveral times.

July 20th, the os uteri was rather more dilated this morning than it had been on the preceding evening, and the membranes were protruding into the vagina. At three o'clock in the afternoon the membranes broke; and upon a very attentive examination it appeared to Dr. Oſborn, and to myſelf, that the diameter from the os pubis to the os ſacrum, hardly, if at all, exceeded one inch and a half. It was therefore determined that the volume of the head ſhould be leſſened. As the patient had paſſed no fæces this day, a clyſter was injected; after the operation of which, Dr. Oſborn evacuated the brain, having firſt made a perforation into the head, which was performed not without ſome difficulty, becauſe it became neceſſary (her belly being very pendulous) to preſs conſiderably upon the abdomen, in order to keep the head over the ſuperior aperture of the pelvis.

She was then left during the night, that putrefaction might begin, and the bones collapſe.

Towards

Towards the morning her pains became violent, accompanied with ſome hyſterical ſpaſms; therefore Dr. Oſborn having picked away great part of the parietal bones with the crotchet, which was by this time practicable, delivered her of the remainder of the head at ſeven in the morning. The body followed with tolerable eaſe, and the placenta came away very favourably.

After her delivery ſhe fell aſleep for ſome hours, but awoke with pain in the abdomen and vomiting. The pain increaſed and continued for ſeveral days, during which time ſhe had evident marks of puerperal fever, which had been moſt probably brought on by the neceſſary force uſed in preſſing upon the abdomen during the labour. She was relieved, however, by bleeding, frequent clyſters, fomentations applied to the abdomen, and the uſe of relaxants. The ſymptoms were at laſt wholly carried off by a miliary eruption, which appeared on her face and neck on the 28th of July; immediately after which ſhe began to regain her ſtrength, and was diſcharged perfectly well on the 8th of Auguſt.

### CASE II.

About the middle of September laſt I was deſired to attend Mrs. Weſt, aged thirty-two, in her labour, which ſhe expected would be near the end of October. She had paſſed the former part of her pregnancy with tolerable eaſe, but towards the expiration of it ſhe had become very thin and uneaſy, from the bulk of her belly, which was become extremely pendulous over the os pubis.

In the very early part of her life ſhe had ſuſtained the misfortune of fracturing the tibia of one ſide, and not long afterwards the femur of the other. The weakneſs ariſing from the confinement neceſſarily occaſioned by theſe accidents, ſuperadded, probably, to a previous diſpoſition to the diſeaſe, produced the rickets; the conſequence of which was, that every bone which ſupported any weight, yielded to the ſuperincumbent preſſure. Her growth by this means was impeded ſo much, that ſhe now only meaſures thirty-nine inches and an half in height. There was every reaſon, therefore, to apprehend, that her labour would be difficult; but I was encouraged to hope for ſucceſs from the event of the caſe of Elizabeth Sherwood *; and of that which I had ſo lately ſeen under the care of Dr. Oſborn; and although her height was leſs than either of them, yet I hoped that her pelvis might not be ſo ſmall.

She was ſeized with regular periodical pains on Wedneſday, Nov. 2, 1785, which conti-tinued through the two following days: but becoming more frequent, and ſtronger, on Saturday, I was ſent for at four o'clock in the afternoon, when I found the os uteri lying very high, and dilated to the ſize of a half crown. By eight o'clock it was dilated to the ſize of a crown, and the membranes protruded, but no part of the child could be felt. The projection of the os ſacrum was, however, immediately diſtinguiſhable, making a large maſs.

I intreated the favour of Dr. Oſborn to ſee the patient, and to give me his opinion of the caſe. He readily complied with my requeſt; and we met at about eleven o'clock. Upon taking a very accurate examination, with a view of aſcertaining the exact dimenſions of the pelvis, Dr. Oſborn accidentally ruptured the membranes, and then we could diſcover

* Vide Dr. Oſborn's Eſſay on laborious Parturition.

 the the head lying above the brim of the pelvis, and were of opinion that the diameter was lefs than an inch and half from the os pubis to the upper part of the os facrum. It was, neverthelefs, (in conformity to the principle which had been laid down by Dr. Ofborn, and which had fucceeded in the cafe of Eliz. Sherwood, where the dimenfions were fmaller) determined to perforate the head. Dr. Ofborn compreffed the abdomen, fo as to keep the head fteady, whilft I perforated it at the lambdoidal future, which Dr. Ofborn had difcovered when the membranes broke, and pointed out the fituation of to me. Having made the opening as large as the great degree of deformity would permit, I evacuated as much of the brain as I could, with the fmall end of a fpoon. Having done this, and having defired the woman to remain as much as was poffible, in an erect pofture, to favour the evacuation of the brain; we left her during the night, to wait for the full effect of the labour-pains, and of any difpofition to putrefaction, which might come on, in order that the head might collapfe more eafily. Her pains were exceedingly ftrong and frequent during the night; and I was again called to her her at fix o'clock in the morning (November 6), when I found that more of the brain had been difcharged in the night, which had diminifhed the bulk of the head, part of which was beginning to enter the fuperior aperture of the pelvis : but the whole bafis of the fkull was ftill above the brim. I introduced the blunt hook into the opening which I had made, and endeavoured to bring the head along, but found it not practicable by fuch a degree of force as I thought to be confiftent with the fafety of the patient. I next attempted to pick away part of the parietes of the fkull ; but, although in the night they had loft confiderably of their firmnefs, they adhered very ftrongly to the dura mater, and to the integuments on the outfide. I therefore, partly by my fingers, and partly by the blunt hook, feparated them from the bones, by which means I think that I gained fome advantage, becaufe I was now able to bring away part of the bones compofing the fides of the cranium. Having thus diminifhed confiderably the bulk of the head, I got the blunt hook firmly fixed in the foramen magnum, and by a regular, commanded force, refting at intervals, I was enabled to deliver the head in about twenty twenty minutes, or half an hour. The body, as it allowed of more compreſſion, came away comparatively with eaſe, and the placenta followed in about ten minutes.

About two hours after her delivery ſhe fell into an hyſterical convulſion, owing, perhaps in ſome meaſure, to the fatigue of her labour, and probably alſo to twenty drops of tinctura thebaica which ſhe had taken. In the courſe of the day ſhe had ſome ſlight degree of pain in the belly. She was ordered to take the ſaline mixture every four hours, with Hoffman's anodyne liquor.

Nov. 7. She had ſlept in the night, and the pain in the abdomen was eaſier this morning. Her pulſe was frequent to 116, but ſhe has naturally a frequent pulſe; and ſhe had no other ſymptoms of fever. She complained, this morning, of ſome pain in making water. She was ordered to take a laxative medicine, which operated three times.

Nov. 8. She had ſlept well in the night. The pain in the abdomen, and that on making water, were much better this morning. Her tongue had a natural appearance, and ſhe had no heat nor thirſt. At night, when I called upon upon her, I found her fitting by the fire whilft her bed was making.

Nov. 9. She repeated her opening medicine and was ftill mending.

Nov. 10. She informed me this morning, that fhe was free from all complaints; and fhe fat up for an hour at night without fatigue.

From this time fhe had no complaints of any kind. During the whole of her confinement fhe lived upon vegetable food, and puddings, and took a laxative medicine every fecond day *.

Such were the circumftances attending thefe two cafes, upon which I have no other remark to make, than that in neither of them was the delivery attended with any great degree of difficulty; nor did the management of them require any extraordinary fkill or dexterity. The great intention of bringing them forward, is to prove that it is poffible to deliver a child, when the head is leffened, through almoft any pelvis, however fmall its dimenfions may be; and therefore, that the Cæfarean operation can

* Mr. Earl, a very intelligent ftudent of Phyfic, did me the favour of his prefence during the delivery of this patient, and vifited her with me through the whole courfe of her confinement.

hardly become neceſſary on account of the diminution of the capacity of the pelvis.

*Chancery Lane,*

Dec. 30, 1785.

